# Supplementary material for: Assessment of the Effectiveness, Socio-Economic Impact and Implementation of a Digital Solution for Patients with Advanced Chronic Diseases: The ADLIFE Study Protocol
Source: Int J Environ Res Public Health. 2023 Feb 10;20(4):3152. doi: 10.3390/ijerph20043152 (PMC9966680; doi:10.3390/ijerph20043152)
Supplement: Supplementary file 1 [file ijerph-20-03152-s001.zip › Supplementary File S1.pdf]

Description of Standard of Care (SoC) across pilot sites

Osakidetza, Basque country

In primary care, assistance is either face-to-face or at home, and sometimes by telephone. In the hospital, care is delivered face-to-face (in some cases, with specific consultations, monographic of a certain pathology-COPD) and by telephone, as well as with the intervention of the advanced practice nursing; closer follow-up is done. In one of the centers there is a telemedicine program for the control and monitoring of patients with COPD who are re-admitted, as well as rehabilitation.

The physical dimension is addressed in a more global way from primary care, while specialists need to have access to that more global assessment carried out in primary care. Treatment is also monitored.

As for the psychological dimension, less assessment is carried out than it should be, also taking into account that patients demand it; there is a lack of psychologists in the health system and therefore the response of the system is slow to these symptoms. In primary care, medication is monitored and in specialist care some psychological health assessment tools are also used (in the specific case of COPD, for example, COPD Assessment Test (CAT) is used) and psychological support is provided in specific situations.

The socio-health aspect is the great forgotten one and yet it is very important, given that it sometimes generates admissions and avoidable consultations. From primary care there is communication with the social services and an annual register is made of interventions of this type. Acute specialized care calls for a screening of the social situation of the patients, which does exist in the medium-stay centres; and in this assessment, the role of the caregivers should also be included. It is proposed to give greater prominence to patient associations, which bring patients and caregivers together, and offer services that are highly valued, such as support groups or talks.

The existential dimension is addressed only when there are situations of terminal care or in very specific cases, and the follow-up is more systematic if the patient has signed the advance directive.

There is communication and coordination between professions, both within the same level of care and between primary and specialized care, and for this purpose different tools are used, such as video calls, telephone, non-face-to-face consultations, although more tools are needed to promote this communication or for those that exist to function correctly.

Odense University Hospital-OUH, Denmark

Patients with COPD who are discharged from care following an exacerbation, and need care consecutively, can get telehealth counselling solutions with nurses for 7-10 days after discharge.

A tablet combined with wireless pulse oximetry and spirometry is used for video consultations. There is a well-functioning collaboration with an external supplier regarding delivery, training, pick-up, cleaning, and calibration of the device. There is also good technical support.

The collaboration between the different actors in health care varies a lot. There is a general lack of cross-specialty interest within this area. The doctors are mostly solving different ad hoc tasks but are not interested in the telehealth solution because there is a lack of evidence on the subject. After the first telehealth counselling days, the commune usually is informed, in consent with the patient. The commune also has COPD nurses in their organization, who will assess whether care should proceed in their organization after the telehealth session.

Assuta Ashdod Hospital together with Maccabi Healthcare Services Southern Region, Israel

The patients who were interviewed were, at the time for the interview, hospitalized in the Maccabi Integrated Care Unit in Assuta Ashdod Hospital. Carers were family members of patients who were or had been recently hospitalized in Assuta Ashdod Hospital. Professional staff of Assuta and Maccabi participated in focus groups, including hospital doctors (internal medicine, cardiology, pulmonology, and emergency medicine), family physicians, nurses, a social worker, a physical therapist, a nutritionist and IT staff. Overall, both patients and carers clearly perceived the main locus of care to be in the community and in the hands of the family doctor. Convalescence care is available after hospitalization.

There is a level of data exchange between the hospital and the community with some limitations. The doctors in the hospital can access selected medical information on a hospitalized patient or a patient being treated in an outpatient clinic in two ways. Through the Maccabi Portal or through the National Electronic Health Record (EHR) exchange. Thus, a doctor (or senior nurse) in the hospital can access information from the patient's community Electronic Medical Record (EMR) including diagnoses, test results and medications. Likewise, the family physician is notified as a pop-up in his EMR when his patient is discharged from the Emergency Room (ER) or hospitalized. He also gets a copy of the ER and hospital discharge summary.

A characteristic of the Israeli system is that there are many specialists who have clinics in the community and consequently, family doctors will frequently refer their patients to these specialists, rather than to refer then to the specialist in the outpatient department of the hospital. These specialists are all using the Maccabi EMR and consequently, all the data entered by specialists in the EMR, including text, is transparent and available to the family doctor.

Maccabi owns a network of complementary medicine clinics. Because of the multicultural nature of the Israeli population, Maccabi is very sensitive to and adapts its services and how they are delivered to the specific cultural and spiritual needs of its diverse populations. For example, they have a translation service that can be accessed during a visit if language is a barrier. Another example, the ultraorthodox population requires same sex clinicians, and modest dress so services for these populations adhere

to these strictures. Maccabi has cultural coordinators for these subpopulations who make sure that there is awareness, and the special needs and sensitivities of these subgroups are addressed.

Social services are provided by the health or social care system, from the Maccabi Social Worker. The National Social Security Institute is responsible for the provision of domiciliary services (home care) as well as pensions and subsidies.

Gesunder Werra-Meißner Kreis-GWMK, Germany

There is communication and interaction between family doctors and specialists in the county. The interaction of medical care with other relevant actors can still be improved in the district, there is a lack of awareness of health care or social services outside the medical sector. Neither the actors know each other nor do those affected know the actors they can turn to.

Most care services in the county are sparsely populated, and care providers work as lone wolfs in the outpatient sector. There are pneumologists in the district, but too few, which means that some patients must turn to pneumologists outside the county. Alternative treatment options are not or only to a limited extent offered on an outpatient basis. A further gap in care is reflected in the lack of guaranteed mental health care.

University of Stradclyde - NHS Lanarshire, Scotland, United Kingdom

Patients receive care at base level at the primary health care providers. If there is a need for more support more entities are involved. Patients in need of more attentive care are entitled to patient-centered physical and social care in care homes and in hospital settings. General practitioners have limited time to assess the state of social needs of chronic patients. As well, little time is allowed for spiritual needs within context of primary care.

A diversity of competencies presents considerable challenges for effective coordination of activities across the health system. The coordination of primary care is achieved through introduction of interdisciplinary primary care teams to coordinate care pathways, but there is still room for improvement. There is weak coordination between inpatient and outpatient care, including poor access to diagnostics.

Region Jämtland Härjedalen, Sweden

Patients with COPD and/or heart failure have their primary care in health care centres. There should be a solid care contact, one person who coordinates the care, but it's not always the fact. For diagnostics, more advanced tests and during exacerbations, the care is given in the county hospital. Both primary care and hospital use the same EHR system, Cosmic.

The municipality have health care professionals who are responsible for patients with bigger needs, like help with getting dressed, eating, daily hygiene or to move. All eight

municipalities have different EHR systems, no one has the same as primary care/hospital. There is a system for information regarding hospital visits: Cosmic Link. In this system, messages are sent to affected organizations when a patient is enrolled/discharged from hospital. The organizations/professionals are added manually. Patients can read their EHR in a site called 1177.se which is national. Here, health information is given, and all inhabitants are told to look for advice here (web site/call center) first, then primary care, and only in emergencies: the ER.

Another branch of the municipality is responsible for social services. This is under another jurisdiction, and sometimes the health care and social care services are inconsistent. The responsibilities of each health care organization in the county are described in an agreement which is often debated: there is a risk that managers are more into economical boarders than what is best for the individual patient.
