# Supplementary material for: Assessment of the Effectiveness, Socio-Economic Impact and Implementation of a Digital Solution for Patients with Advanced Chronic Diseases: The ADLIFE Study Protocol
Source: Int J Environ Res Public Health. 2023 Feb 10;20(4):3152. doi: 10.3390/ijerph20043152 (PMC9966680; doi:10.3390/ijerph20043152)
Supplement: Supplementary file 1 [file ijerph-20-03152-s001.zip › Supplementary File S2.pdf]

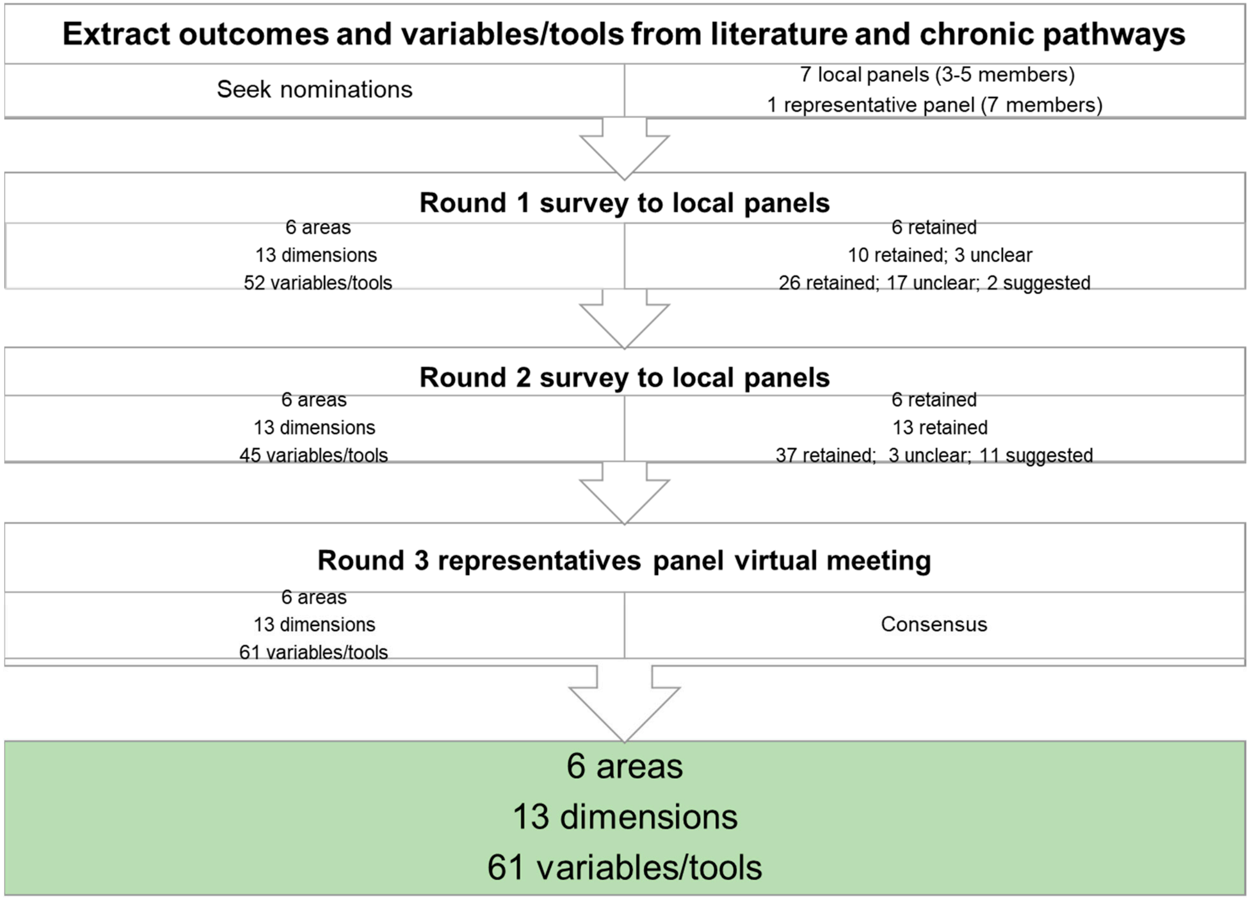

Figure S1. Workflow of the three-round modified Delphi process for consolidating the health-related outcome set

Table S1. List of variables/tools proposed to be used to monitor outcomes of care

ADLIFE Area	ADLIFE dimension	Outcome measurement
Symptoms, functioning, quality of life	Autonomy, control	Dementia (yes/no), JITAI, caregiver support, EQ-5D-5L, Adherence to care plan
	Symtom control	Visits to primary care/nurse, self-assessment methods/condition specific standard PROMs (such as physical activity, blood pressure, weight, sputum), EQ-5D-5L
	Mood and emotional health	HADS, PHQ2
	Social context	EQ-5D-5L
	Activities of daily living	Lawton scale, Barthel index, KCCQ or CAT (condition specific), walking distance (6-min walk), smoking (yes/no), peak oxygen consumption, Gijon scale
Disutility of care	Polypharmacy	Prescribed drugs (code, dosage, frequency, length of treatment)
	Appropriateness	De-prescription, Treatment side effects, Comparison between prescribed drugs and recommended drugs (adherence to recommendations)
Quality of death	Place of death	Date of death, desired and actual place of death
	Advances directives	Registered document, annotated preference of care, identified for palliative care
Clinical status	Patient attention time	Visits: GP, specialized care, nurse, hospital admissions and stays, unplanned A&E, cause of hospital admissions (COPD or HF)
	Stability (undesired events)	Admissions in iCU, home oxygen therapy (yes/no), extra inhalation medication, AECOPD episodes (yes/no), HF decompensation, sputum and color, cough, need of extra diuretics, weight

**ADLIFE Area**	**ADLIFE dimension**	**Outcome measurement**
	Complexity (ie hurdle, severity)	Age, sex, active diagnoses, Charlson index, FEV1, Ejection fraction evolution, NYHA, ACCF/AHA, CAT, Gold Scale, mMRC, use of Non-Invasive Ventilation (NIV)
	Side effects	Lab results (haemoglobin, sodium, potassium, platelets, creatinine, urea), eGFR, TP InR, proBNP, glycemia, HbA1c
Healthcare responsiveness	Participation	Shared decision making, ask 3 questions (options, pros/cons, likelihood), Perceived participation
	Continuity of care	Perceived communication and coordination
Care	Satisfaction	Overall Job Satisfaction scale (SEHC), person-centered climate questionnaire - PCQ-S
	Carer burden	Wellbeing questionnaire, perceived quality of care

Table S2. Correspondence with ICHOM standard sets

ADLIFE Areas	ADLIFE Dimensions	HF ICHOM Dimensions	Older person ICHOM Dimension
Symptoms, functioning quality of life	Autonomy, control	✓ (Independence)	✓
	Symptom control	✓	
	Mood and emotional health	✓ (Psicosocial Health)	✓
	Social context		✓ (Loneliness and isolation)
	Activities of daily living	✓	✓
Disutility of care	Polypharmacy		✓
	Appropriateness		
Quality of death	Place of death		✓
	Advances directives		

ADLIFE Areas	ADLIFE Dimensions	HF ICHOM Dimensions	Older person ICHOM Dimension
Clinical status	Patient attention time	✓ Hospital visits	✓ Time spent in Hospital
	Survival (quality adjusted)		✓
	Complications (i.e hurdle, severity)	✓	
	Side effects	✓	
Healthcare responsiveness	Participation		✓ Participation and decision making
	Continuity of care		
Care	Satisfaction		
	Carer Burden		✓
